# Health morbidities in carers with experience of domestic violence and abuse

**DOI:** 10.1007/s00127-025-02959-4

**Published:** 2025-07-16

**Authors:** Emilie K. Wildman, Hannah Dickson, Deirdre MacManus, Sally McManus, Elizabeth Kuipers, Juliana Onwumere

**Affiliations:** 1 Department of Psychology, Institute of Psychiatry, Psychology and Neuroscience, King’s College London, 16 De Crespigny Park, Denmark Hill, SE5 8AF London, UK; 2 Department of Forensic and Neurodevelopmental Sciences, Institute of Psychiatry, Psychology and Neuroscience London, King’s College London, London, UK; 3 Violence and Society Centre, City St George’s, University of London, London, UK; 4 National Centre for Social Research, 35 Northampton Square, London, UK; 5 Bethlem Royal Hospital, South London and Maudsley NHS Foundation Trust, Beckenham, UK

**Keywords:** Carers, Domestic violence, Mental health, Physical health

## Abstract

**Background:**

The poor health of unpaid carers is well-documented. Evidence also highlights that carers can experience high levels of domestic violence and abuse (DVA). However, links between DVA victimisation and health outcomes in carers remains largely overlooked. We examined DVA prevalence in carers and non-carers, and the relationship between carers’ DVA experience and health morbidities.

**Methods:**

We analysed data from a general population probability sample survey of 6,971 adults (aged ≥ 16 years) in England. Multivariable logistic regression models examined associations between caregiving, DVA experience, and mental and physical health morbidities (i.e., common mental disorders (CMD), probable post-traumatic stress disorder (PTSD), harmful alcohol use and chronic physical health conditions), adjusting for demographic and socioeconomic factors.

**Results:**

One person in five reported caring responsibilities. Caring was associated with higher odds of CMD and chronic physical health conditions. One in three carers reported experiencing DVA in adulthood, and carers were more likely to be victims of DVA than non-carers. In carers who experienced DVA, compared to carers reporting no DVA, adjusted odds of CMD (aOR 2.88, 95% CI 2.11–3.95); probable PTSD (aOR 5.67, 95% CI 3.12–10.30); hazardous alcohol use (aOR 1.53, 95% CI 1.09–2.15) and chronic physical health conditions (aOR 1.53, 95% CI 1.14–2.06), were significantly higher.

**Conclusions:**

The risk of DVA victimisation among carers and the associated vulnerability to poorer health outcomes were highlighted. The need for greater awareness and identification of carers’ risk of DVA, and better provision of support for the negative health consequences are emphasised.

## Introduction

Domestic violence and abuse (DVA) is a global public health problem [1] with negative health implications for the victimised, including elevated levels of depression, post-traumatic stress symptoms, alcohol and substance misuse and physical health conditions [2–4]. In the United Kingdom (UK), the definition of DVA comprises different types of violent behaviours (e.g., physical, sexual, psychological, and coercive controlling), involving intimate partners and/or relatives where both are aged sixteen and over [5]. In the year ending March 2023, 889,441 police recorded DVA-related offences were reported across England and Wales with approximately 700,000 recorded as violent offences against the person (Office for National Statistics (ONS) [6]). This figure likely underestimates the true scale of the problem since victims are less likely to report DVA incidents to the police [7].When DVA goes undisclosed by victims and undetected by professionals, opportunities to support victims and prevent escalations in violence are missed [8,9].

Domestic homicide reviews in the UK highlight the dangers of missing service-level opportunities to intervene and support victims [10]. They also frequently highlight the role of informal (unpaid) caregiving responsibilities. Informal (unpaid) carers (referred to hereafter as carers) have been identified as perpetrators and, more often, victims of domestic homicides [10]. However, carers’ experiences of DVA have remained largely overlooked and poorly understood [11, 12]. For example, we lack a broader understanding of the scale of the problem in the adult general population, and whether experiencing DVA contributes to the poorer health outcomes observed in carers compared to non-carers. This remains an important issue given approximately 1 in 5 (i.e., 10.58 million) adults are providing informal (unpaid) care across the UK, with approximately 6 million living in England and Wales [13]. Informal carers provide care and support in an unpaid capacity to a relative, friend, or significant other living with an illness, disability or care needs related to older age. Females compared to male peers are more likely to assume informal carer roles. Carers significant contributions to health and social care systems are widely recognised and their economic value is recorded at £160 billion per annum [13].

A caregiving role has been associated with different negative physical (e.g., diabetes, obesity, hypertension, pain-related conditions), and mental health impacts (e.g., care burden, psychological distress and common mental disorders) [14–16]. Several studies have highlighted experiences of trauma and post-traumatic stress symptoms in carers of individuals with different conditions including autism spectrum disorders [17]; psychosis [18], and cancer [19]. Data on modifiable health-risk behaviours in carers (e.g., alcohol use), are limited [20]. However, we do have some data to suggest carers’ vulnerability to health risk behaviours. For example, if we focus on alcohol consumption, increased risks of hazardous alcohol use are recorded in carers of individuals with mental illness [21],

Alzheimer’s disease and dementia [22], and other chronic illnesses [20]. An improved understanding of the health morbidities of carers, and factors (e.g., DVA experience) that may influence their health-related outcomes offers a helpful pathway to inform and tailor potential support interventions (National Institute for Health and Care Excellence (NICE), [23]).

This study sought to investigate the health morbidities of carers in a representative sample of the adult population of England and explore the relationship with carers’ DVA experiences. We hypothesised that: (a) carers would report significantly greater mental and physical health morbidity compared to non-carers; (b) the prevalence of experiencing DVA would be greater in carers than non-carers; and (c) carers reporting a history of DVA experience would report greater mental and physical health morbidities compared to carers reporting no history of DVA. We also explored whether experience of DVA would account for some of the difference in recorded morbidity, between carers and non-carers. As this was exploratory, no a priori hypothesis was stipulated.

## Method

### Participants and procedures

The study comprised secondary analysis of cross-sectional data from the 2014 Adult Psychiatric Morbidity Survey (APMS) [24]. The survey was approved by the West London National Research Ethics Committee (14/LO/0411) and permission to use the data in our study was granted by NHS Digital.

The APMS is administered once every seven years, and the current data reflects the most recent in the series. The APMS uses a multistage stratified random sampling design, to provide data on the prevalence of treated and untreated psychiatric conditions in adults, aged ≥ 16, living in private households in England. Full details of the APMS methodology have been extensively published elsewhere (e.g [25]). APMS data collection involved trained interviewers administering the survey using computer-assisted interviewing. Self-completion modules were used to enable greater privacy for collection of more sensitive data.

Overall, 7,546 participants were interviewed (57% of those potentially eligible to participate). To produce a sample representative of the national population, data were weighted to account for selection probability and nonresponse.

### Measures

#### Exposures

To identify carers, participants were asked whether they provide informal (unpaid) support to relatives, friends, neighbours, or others, due to long-term physical or mental ill-health, or age-related difficulties. A binary variable was derived, coding non-carers as 0 and carers as 1. Details regarding the average number of caregiving hours per week, the carer’s relationship to the care recipient, and living situation of the carer and care recipient were also recorded to provide contextual information about the nature of caregiving within the sample.

The self-completion section of the interview asked participants about their experiences of DVA from relatives and partners/spouses during adulthood (i.e., since aged sixteen years). This included: physical violence (e.g., being kicked, bitten, and/or hit); emotional and psychological violence, and control and coercion (EPC) (e.g., being repeatedly belittled, frightened, and/or prevented from seeing friends/relatives); and sexual violence (e.g., sexual contact without consent). Follow-up questions also established whether participants had experienced DVA during the 12 months preceding the interview. Binary variables were derived to indicate the absence or presence of each type of DVA and of any DVA, experienced in adulthood and the past year, respectively.

#### Outcomes

Common mental disorders (CMD) were assessed using the Clinical Interview Schedule-Revised (CIS-R) [26], which measures symptoms in the past week to provide estimates of six CMDs, according to ICD-10 criteria. Scores range from 0 to 57, with scores ≥ 12 indicating the presence of clinically meaningful symptoms [27].

Probable PTSD was assessed using the PTSD Checklist-Civilian Version (PCL-C) [28], which assesses the experience of symptoms in the preceding month and provides a score ranging from 17 to 85. Consistent with the scoring used in the original APMS report [29], probable PTSD was considered present in participants scoring ≥ 50, and/or meeting Diagnostic Statistical Manual (DSM-IV) criteria for PTSD.

Harmful alcohol use was measured using the Alcohol Use Disorders Identification Test (AUDIT) [30]. Scores range from 0 to 40, and the accepted threshold of ≥ 8 was taken to indicate the presence of hazardous alcohol use, based on the validation [31], and recent literature [32].

Participants were shown a card with 20 physical health conditions. The list comprised: cancer; diabetes; epilepsy; migraine; stroke; heart-attack; high blood pressure; bronchitis; asthma; allergies; arthritis; infectious diseases; cataracts/eyesight, hearing, bowel/colon, muscular, digestive, liver, bladder, and skin problems. A condition was classified as present if participants reported that it: (a) had been formally diagnosed by a health professional; and (b) was present in the 12 months preceding interview.

A binary variable was derived for each outcome, to indicate its absence (coded as 0) or presence (coded as 1).

#### Covariates

Demographic questions established participants’ self-reported sex (male, female), age band(years) (i.e.,16–24, 25–34, 35–44, 45–54, 55+), and marital status (single; married/cohabiting; separated/divorced/widowed). Ethnicity was self-defined based on UK census categories (ONS, 2011), and further categorised into: White British, White other, Black/African/Caribbean/Black British/Asian/Asian British, and Mixed/Multiple/Other. For some analyses, ethnicity was further grouped into White British and other, due to relatively small numbers of participants in all other categories. Socioeconomic context was captured from participants’ housing tenure (owner-occupier; renting from a social, or private landlord), employment status (employed, unemployed, economically inactive), and past-year debt (participants reported if they had been seriously behind paying for bills and loans in the past year, with a binary variable to indicate the absence or presence of debt).

Adverse life events in adulthood (i.e., occurring after aged 16 years) were assessed using the List of Threatening Experiences (LTE) [33]. Two binary variables were constructed, reflecting the experience of personal (e.g., bereavement, serious interpersonal difficulties), and material events (e.g., job loss, major financial crisis), respectively. Serious illness in a close relative, violence in the home, and sexual abuse were excluded from the list as potentially being confounders in the association between caregiving, experience of DVA and health morbidities.

### Statistical analysis

All analyses were conducted in STATA v17 [34] using ‘survey’ (svy) commands. We used weighted data to take account of the complex survey design, selection probabilities and non-response. Group differences in sociodemographic and socioeconomic circumstances were assessed with uncorrected chi-square and design-based ANOVA tests. To investigate health morbidities in carers compared to non-carers, we ran separate multiple variable logistic regression models for each binary-coded outcome (i.e., CMD, probable PTSD, hazardous alcohol use, chronic physical health conditions). Models 1 examined crude associations between caregiving and outcomes. Models 2 were adjusted for demographic and socioeconomic confounders and identified a priori from the existing literature and confirmed in analyses (i.e., age, sex, ethnicity, marital status, employment status, housing tenure, and debt). Multicollinearity was assessed and found not to be a problem, with variance inflation factor < 1.5 for all variables.

The prevalence rates of DVA reported by carers and non-carers, were calculated and tabulated. Significant differences in prevalence were assessed; crude and adjusted odds ratios, 95% confidence intervals and *p*-values are reported. To investigate whether carers with a history of DVA reported greater health morbidities compared to carers reporting no DVA, a subgroup analysis was performed. We conducted logistic regression models (as described above), using a binary exposure variable to indicate the absence or presence of any DVA experience in adulthood. To explore to what extent, if any, experiencing DVA accounted for some of the difference in morbidity between carers and non-carers, we conducted exploratory tests for the modifying effect of DVA. An interaction term (i.e., testing for an interaction between caregiving and experience of DVA in adulthood), was added to logistic regression models, and was included if there was evidence of an interaction. If there was no evidence of an interaction, we adjusted models 2 to account for adulthood DVA experience (models 3), and other personal and material adverse life events in adulthood (models 4), to test whether the relationship between caregiving and the outcomes diminished as a result of their inclusion.

## Results

6% of participants (*n* = 462) did not complete any of the self-completion section of the interview (i.e., non-completers) and were excluded from analyses. Compared to completers, non-completers were more likely to be older (F(1, 352) = 91, *p* < 0.0001), widowed (χ² = 60.4, df = 2, *p* < 0.0001), social renters (χ² = 36.4, df = 2, *p* < 0.0001), and economically inactive (χ² = 108.4, df = 2, *p* < 0.0001). Analyses were conducted on the 6,971 participants who provided information on all exposures, outcomes, and covariates. Actual counts are reported throughout, together with survey-weighted proportions and 95% confidence intervals. Alpha levels of < 0.05 were considered significant.

### Profile of carers

Table 1 details the demographic, socioeconomic, and health morbidity profile of participants. One in five participants reported caregiving responsibilities. Carers were more likely to be older than non-carers (F(1,352) = 71, *p* < 0.0001) with a greater proportion self-classified as female (χ²=12.7, df = 1, *p* < 0.001), White British (χ² = 25.8, df = 4, *p* < 0.005), married (χ² = 46.6, df = 2, *p* < 0.0001), economically inactive (χ² = 37.8, df = 2, *p* < 0.0001), and in debt in the year preceding the interview (χ² = 6.7, df = 1, *p* < 0.05). A greater proportion of carers reported experiencing one or more personal (χ² = 69.7, df = 1, *p* < 0.0001), and material (χ² = 17.9, df = 1, *p* < 0.0005) adverse life events in adulthood, respectively.

**Table 1:** Demographic, socioeconomic and health morbidity profile of participants by caregiving status and experience of DVA^a^

	Total (%)		Non-carers (%)		Carers (%)		*p*-value	Non-carers no DVA (%)		Non-carers with DVA (%)		Carers no DVA (%)		Carers with DVA (%)		*p*-value
**Gender** Male Female	2,843 4,128	(49.2) (50.8)	2,285 3,200	(50.3) (49.7)	558 928	(45) (55)	**<0.005**	1,737 2,122	(53.1) (46.9)	548 1,078	(42.7) (57.3)	429 524	(52.0) (48.0)	129 404	(31.5) (68.5)	**<0.0001**
**Age**, mean (SD)	46.7	(18.7)	45.6	(19.0)	50.7	(16.9)	**<0.0001**	46.5	(19.4)	43.2	(17.5)	52.9	(16.7)	46.4	(16.4)	**<0.0001**
**Age** 16 – 24 25 – 34 35 – 44 45 – 54 55+	529 987 1,121 1,224 3,110	(14.3) (17.2) (16.4) (17.8) (34.4)	458 866 909 887 2,365	(15.6) (19.2) (16.8) (16.2) (32.1)	71 121 212 337 745	(9.3) (9.4) (15.0) (23.5) (42.8)	**<0.0001**	316 582 596 558 1,807	(15.5) (19.0) (15.7) (15.4) (34.4)	142 284 313 329 558	(16.0) (19.8) (19.7) (18.5) (26.0)	36 62 110 207 538	(7.8) (8.2) (12.5) (23.1) (48.5)	35 59 102 130 207	(12.1) (11.8) (19.9) (24.4) (31.8)	**<0.0001**
**Ethnic group** White British White other Black/African/Caribbean/Black British Asian/Asian British Mixed/Multiple/Other	5,959 392 172 317 131	(81.3) (6.6) (2.9) (6.8) (2.4)	4,651 336 136 257 105	(80.3) (7.3) (3) (7.0) (2.4)	1,308 56 36 60 26	(85.0) (3.9) (2.8) (6.0) (2.3)	**<0.005**	3,256 251 90 192 70	(79.6) (7.7) (2.9) (7.5) (2.4)	1,395 85 46 65 35	(82.3) (6.4) (3.2) (5.7) (2.4)	846 35 21 39 12	(86.2) (3.9) (2.6) (5.6) (1.7)	462 21 15 21 14	(82.7) (3.7) (3.3) (6.8) (3.4)	**<0.05**
**Marital Status** Married/Cohabiting Single Separated/Divorced/ Widowed	3,901 1,460 1,610	(62.6) (24.1) (13.2)	2,957 1,217 1,311	(60.7) (25.8) (13.4)	944 243 299	(69.7) (17.8) (12.5)	**<0.0001**	2,231 799 829	(63.1) (25.0) (11.9)	726 418 482	(54.5) (28.0) (17.5)	664 134 155	(74.7) (15.6) (9.7)	280 109 144	(60.1) (22.0) (17.9)	**<0.0001**
**Employment** Employed Unemployed Economically inactive^b^	3,833 201 2,937	(61.3) (3.4) (35.4)	3,071 154 2260	(63.1) (3.3) (33.6)	762 47 677	(54.4) (3.5) (42.1)	**<0.0001**	2,101 87 1,671	(62.3) (2.9) (34.9)	970 67 589	(65.2) (4.5) (30.3)	471 22 460	(52.9) (2.5) (44.6)	291 25 217	(57.4) (5.4) (37.2)	**<0.0001**
**Housing Tenure** Owner-occupier Social renter Private/other renter	4,618 1,120 1,233	(64.5) (15.1) (20.3)	3,580 856 1,049	(63.5) (14.2) (22.3)	1,038 264 184	(68.5) (15.1) (20.3)	**<0.0001**	2,718 495 646	(67.2) (12.5) (20.3)	862 361 403	(53.5) (19.1) (27.4)	725 139 89	(73.8) (16.1) (10)	313 125 95	(58.3) (23.1) (18.6)	**<0.0001**
**Past-year debt** Not in debt In debt	6,443 528	(92.9) (7.1)	5,092 393	(93.3) (6.7)	1,351 135	(91.3) (8.7)	**<0.05**	3,701 158	(95.8) (4.2)	1,391 235	(86.7) (13.3)	900 53	(94.6) (5.4)	451 82	(85.1) (14.9)	**<0.0001**
**Number of close contacts** mean (SD)	13.6	(10.1)	13.4	(9.5)	14.6	(12)	**<0.005**	14.1	(9.6)	11.5	(8.9)	16.3	(13.1)	11.2	(8.1)	**<0.0001**
**Perceived social support** mean score (SD)	20.2	(1.9)	20.2	(1.9)	20.1	(1.9)	ns	20.4	(1.5)	19.8	(2.5)	20.3	(1.7)	19.8	(2.3)	**<0.0001**
**Personal ALE** None One or more	1,019 5,952	(19.2) (80.8)	890 4,595	(21.2) (78.8)	129 1,357	(11.5) (88.5)	**<0.0001**	734 3,125	(24.4) (75.6)	156 1,470	(12.6) (87.4)	89 864	(12.1) (87.9)	40 493	(10.5) (89.5)	**<0.0001**
**Material ALE** None One or more	3,380 3,591	(48.9) (51.1)	2,702 2,778	(50.2) (49.8)	673 813	(44.0) (56.0)	**<0.0005**	2,108 1,751	(54.8) (45.2)	599 1,027	(37.9) (62.1)	468 485	(47.7) (52.3)	205 328	(36.6) (63.4)	**<0.0001**
**Scores on outcome measures**																
**CIS-R**, mean (SD)	5.2	(7.4)	4.9	(7.2)	6.6	(7.9)	**<0.0001**	3.6	(5.5)	8.3	(9.8)	4.9	(6.5)	9.8	(9.5)	**<0.0001**
**PCL-C**, mean (SD)	25.1	(10.8)	24.9	(10.7)	26	(11.1)	**<0.005**	22.7	(8.2)	30.5	(14.2)	23	(7.7)	32	(14.1)	**<0.0001**
**AUDIT**, mean (SD)	4.5	(4.7)	4.6	(4.7)	4.1	(4.4)	**<0.001**	4.3	(4.2)	5.2	(5.8)	3.9	(3.9)	4.4	(5.4)	**<0.0001**
**Physical health conditions**, mean (SD)	1.4	(1.6)	1.3	(1.6)	1.7	(1.7)	**<0.0001**	1.2	(1.5)	1.5	(1.8)	1.6	(1.6)	1.9	(1.9)	**<0.0001**

ALE = adverse life events; AUDIT = Alcohol Use Disorders Identification Test; CIS-R = Clinical Interview Schedule-Revised; DVA = domestic violence and abuse; ns = not significant; PCL-C = PTSD Checklist-Civilian Version; SD = standard deviation

^a^ Participants are grouped according to their caregiving status, and whether or not they reported any experience of DVA in adulthood

^b^ Economically inactive comprises participants who are: unable to work due to disability/long-term illness; students; retired; looking after the home

In terms of the relationship between carers and care recipients, most were caregiving for a parent (43.1%), followed by caring for a: partner/spouse (15.1%); non-relatives (13.4%); children (12.7%); other relatives (10.9%); and siblings (4.9%). 36% (*n* = 411) reported co-residing with the care recipient. Co-residence was most likely among carers of partners/spouses, followed by that caregiving for their children. Significantly greater proportions of those caring for partners/spouses and children also reported having the highest number of average caregiving hours per week (hpw) (> 100 hpw) (χ² = 470.9, df = 20, *p* < 0.0001), compared to carers of other groups of care recipients (see supplementary *Table S1* for further details).

### Mental and physical health morbidities in carers

In carers, the odds of CMD were 1.6 times higher than in non-carers (95% CI 1.35–1.90, *p* < 0.0001), and remained higher after adjustment for potential confounders (aOR 1.55, 95% CI 1.29–1.85, *p* < 0.0001). The odds of probable PTSD were not significantly different in carers and non-carers in unadjusted (*p* = 0.454), or adjusted models (*p* = 0.670). Being a carer was associated with lower odds of hazardous alcohol use (OR 0.74, 95% CI 0.62–0.89, *p* < 0.001), relative to being a non-carer. However, this association was no longer significant after adjustment for confounders (*p* = 0.126). The odds of having one or more physical health conditions was 1.67 times higher in carers than non-carers (95% CI 1.44–1.94, *p* < 0.0001), remaining significantly higher after adjustment for confounders (aOR 1.30, 95% CI 1.12–1.52, *p* < 0.001) (see Table 2).

**Table 2:** Associations between caregiving and mental and physical health morbidities

	Models 1^a^			Models 2^b^		
	OR	95% CI	*p*-value	aOR	95% CI	*p*-value
Common mental disorder	1.60	1.35–1.90	**<0.0001**	1.55	1.29–1.85	**<0.0001**
Post-traumatic stress disorder	1.10	0.85–1.42	0.454	1.06	0.81–1.39	0.670
Hazardous alcohol use	0.74	0.62–0.89	**<0.001**	0.87	0.72–1.04	0.126
Physical health conditions	1.67	1.44–1.94	**<0.0001**	1.30	1.12–1.52	**<0.001**

aOR = adjusted odds ratio; CI = confidence intervals; OR = odds ratio

^a^ Models 1: Unadjusted associations between caregiving and outcome variables

^b^ Models 2: Adjusted for age, sex, ethnicity, marital status, housing tenure, employment status and debt

### Carers’ experiences of DVA

One in three carers reported experiencing DVA during adulthood and carers, compared to non-carers, were significantly more likely to be victims of all types of DVA. Following adjustment for confounders carers, compared to non-carers, remained significantly more likely to be victims of physical (aOR 1.27), EPC (aOR 1.40), and sexual DVA (aOR 1.66). No significant differences were identified in reported rates of past-year DVA (see Table 3).

**Table 3:** The experience of DVA in carers and Non-Carers

	Non-carers (%)	Carers (%)	Total (%)	OR	95% CI	*p*-value	aOR^a^	95% CI	*p*-value
Types of DVA^b^									
EPC (adult lifetime)^c^	1,305 (21.4)	429 (27.3)	1,734 (22.6)	1.38	1.19–1.60	**<0.0001**	1.40	1.20–1.63	**<0.0001**
Physical (adult lifetime)	1,059 (17.5)	349 (21.3)	1,408 (18.3)	1.28	1.10–1.49	**<0.005**	1.27	1.09–1.49	**<0.005**
Sexual (adult lifetime)	134 (1.9)	54 (3.4)	188 (2.2)	1.84	1.27–2.67	**<0.001**	1.66	1.12–2.46	**<0.01**
Any DVA (adult lifetime)	1,626 (27.3)	533 (33.9)	2,159 (28.7)	1.37	1.19–1.57	**<0.0001**	1.41	1.22–1.63	**<0.0001**
Any DVA (past year)	271 (5.1)	74 (5)	345 (5.1)	0.98	0.73–1.32	ns	1.10	0.81–1.50	ns

aOR = adjusted odds ratio; CI = confidence intervals; DVA = domestic violence and abuse; EPC = emotional and psychological violence, control and coercion; ns = not significant; OR = odds ratio

^a^ Adjusted for age, sex, ethnicity, marital status, housing tenure, employment status, and debt

^b^ DVA includes violent victimisation from a family member and/or partner/spouse

^c^ Adult lifetime denotes experience of DVA occurring after the age of sixteen

Carers who reported experiencing any DVA in adulthood were also more likely to be female, social renters, unemployed and in debt, compared to carers and non-carers who reported having no DVA experience, as well as non-carers who reported experiencing DVA. Carers who reported experiencing DVA reported higher mean scores across all key outcome measures compared to carers and non-carers reporting no DVA, as well as non-carers who reported experiencing DVA (see Table 1).

### Health morbidities in carers reporting DVA in adulthood

In carers reporting any DVA experience (*n* = 533), the odds of CMD were almost fourfold (OR 3.86), and of probable PTSD were over sevenfold (OR 7.25), compared to carers reporting no DVA (*n* = 953). The odds of hazardous alcohol use were also significantly higher (OR 1.39). After adjustment for confounders, the odds of CMD (aOR 2.88); probable PTSD (aOR 5.67); and hazardous alcohol use (aOR 1.53) remained significantly higher in carers with experience of DVA.

The odds of having one or more physical health conditions were not significantly different in carers with and without DVA experience (*p* = 0.092). After adjustment for confounders, however, the odds were significantly higher in carers who experienced DVA (aOR 1.53), compared to carers reporting no DVA (see Table 4).

**Table 4:** Health morbidities in carers who reported experience of DVA in adulthood versus carers with no DVA experience

	Models 1^a^			Models 2^b^		
	OR	95% CI	*p*-value	aOR	95% CI	*p*-value
Common mental disorder	3.86	2.89–5.14	**<0.0001**	2.88	2.11–3.95	**<0.0001**
Post-traumatic stress disorder	7.25	4.32–12.14	**<0.0001**	5.67	3.12–10.30	**<0.0001**
Hazardous alcohol use	1.39	1.01–1.91	**<0.05**	1.53	1.09–2.15	**<0.05**
Physical health conditions	1.27	0.96–1.68	0.092	1.53	1.14–2.06	**<0.005**

aOR = adjusted odds ratio; CI = confidence intervals; OR = odds ratio

^a^ Models 1: Unadjusted associations between caregiving and health morbidities, in carers with DVA experience in adulthood compared to carers reporting no experience of DVA

^b^ Models 2: Adjusted for age, sex, ethnicity, marital status, housing tenure, employment status and debt

### The role of DVA in the association between caregiving and health morbidities

Exploratory tests of the modifying effect of experiencing DVA were conducted, by adding an interaction term to the logistic regression models. Neither experience of DVA in adulthood nor in the past year were found to be effect modifiers in the association between caregiving and CMD (adulthood *p* = 0.862; past-year *p* = 0.449), probable PTSD (adulthood *p* = 0.086; past-year *p* = 0.128), hazardous alcohol use (adulthood *p* = 0.857; past-year *p* = 0.645), or physical health conditions (adulthood *p* = 0.412; past-year *p* = 0.296).

Logistic regression models investigating associations between caregiving and health morbidities, after adjustment for confounders (models 2), were additionally adjusted to account for DVA experience in adulthood (models 3), and exposure to personal and material adverse life events in adulthood (models 4), to investigate whether the associations between caregiving and health morbidities attenuated because of their inclusion. Table 5 reports the associations between caregiving and outcomes, in regression models 3–4. In the fully adjusted models (models 4), caring remained an independent predictor of CMD and physical health conditions. DVA experience in adulthood also demonstrated significant independent associations with all outcome variables. Further tabulated information for all variables entered logistic regression models are reported in supplementary tables S2-S5.

**Table 5:** Associations between caregiving and mental and physical health morbidities, accounting for experience of DVA and other adverse life events in adulthood

	Models 3^c^			Models 4^d^		
	aOR	95% CI	*p*-value	aOR	95% CI	*p*-value
Common mental disorder	1.44	1.19–1.73	**<0.0001**	1.41	1.17–1.70	**<0.0001**
Post-traumatic stress disorder	0.95	0.72–1.25	0.701	0.92	0.70–1.20	0.531
Hazardous alcohol use	0.85	0.70–1.02	0.079	0.83	0.69–1.00	0.054
Physical health conditions	1.26	1.08–1.48	**<0.005**	1.23	1.05–1.43	**<0.01**

aOR – adjusted odds ratio; CI = confidence intervals; OR = odds ratio

^C^ Models 3: Model 2 (i.e., adjusted for age, sex, ethnicity, marital status, housing tenure, employment status and debt) plus adjustment for DVA experience in adulthood

^D^ Models 4: Model 3 plus adjustment for experience of personal and material adverse life events in childhood

## Discussion

Using a national probability sample survey of the general population, this study examined the prevalence of DVA in carers and non-carers and explored the relationship between carers’ DVA experiences and health morbidities.

### Health morbidities in carers

In partial confirmation for hypothesis 1, following adjustment for confounders, carers had higher odds of CMD and poorer physical health morbidity than non-carers, which offers further support highlighting mental and physical health impacts associated with caregiving [14, 15].Contrary to predictions, however, carers and non-carers did not differ significantly in their likelihood of probable PTSD, although carers’ mean score on the PCL-C highlighted significantly greater symptom severity. Younger carer age has been identified as a risk factor for greater PTSD symptomatology [35].

In our study, carers’ mean age was 50.7 years, and the largest carer group were those in the 55 years + age category. It is possible that a younger participant group could yield a different pattern of findings and that carer age might have served as a protective factor against PTSD risk. Social support can also be protective against PTSD in the general population [36]and in carer samples [35]. While there were no significant differences in levels of perceived social support between carers and non-carers in our study, carers reported a significantly higher mean number of close contacts than non-carers. Thus, carers’ levels of social support may also have mitigated risks of probable PTSD [37]. While previous research has identified high proportions of hazardous alcohol use in carers [20], our findings found no significant differences in the likelihood of hazardous alcohol use between carers and non-carers. Caregiving may also be associated with more responsible (and less problematic) drinking habits for more pragmatic reasons [38]. Increased alcohol use may hinder abilities to manage the responsibilities associated with caregiving [38].

### Carers’ experience of DVA

Confirming hypothesis 2, carers were significantly more likely to have been victims of all types of DVA in adulthood than non-carers, after adjustment for identified confounders. The survey design of fixed response options precludes confirmation of whether the DVA reported by carers had been perpetrated by the care recipient. However, carers’ experiences of DVA may be a direct result of their caregiving role [39]. We do know, for example, that carers drawn from different conditions such as schizophrenia [40] and autism spectrum disorders [41], are more likely to be the targets of care recipient violence than members of the general population. Recent evidence has also highlighted that carers, supporting a range of mental and physical illness groups, often experience care recipient violence [42, 43]. Furthermore, domestic homicide reviews frequently identify caregiving responsibilities as a risk factor for victimisation [10].

If we consider recent DVA exposure, 5% of participants reported past-year DVA experience, but with no significant differences in reports were observed between carers and non-carers. While these rates are comparable to past-year prevalence rates of DVA victimisation of 5.5% across England and Wales [44], we know that socially undesirable behaviours and/or stigmatised experiences are often underreported [45]. Complex family relationships, caregiving responsibilities, and perceived support needs of the perpetrator can further inhibit disclosure in victims of DVA [46]. Given that barriers to disclosure can be further compounded in carers [11], the likelihood of underreporting may have been greater in carers, than non-carers, in our study. The absence of contextual information about the caregiving role prevents a fuller understanding of the type and range of factors that may have contributed to the finding of equivalent past-year DVA rates in carers and non-carers (e.g., [47]). Care recipient violence remains largely understudied and, accordingly, our understanding of risk and protective factors is limited [48]. Within caregiving relationships for individuals with a severe mental health condition such as schizophrenia, however, positive associations between carers’ experiences of care recipient perpetrated violence, co-residency and increased family contact, are well-documented [49, 50]. In our study, almost two-thirds of carers lived separately from the care recipient at the time of interview. Furthermore, the two largest carer groups in our sample (i.e., those caregiving for a parent, and non-relative) reported the lowest frequencies of caregiving hours (i.e., 0–9 hpw). It is therefore possible that living separately from, and less frequent contact with, care recipients may have been protective against the risk of violent victimisation for many carers.

### Health morbidities in carers reporting experience of DVA

Findings confirmed hypothesis 3; with carers reporting any DVA experience in adulthood having significantly poorer outcomes on all measures of mental and physical health, compared to carers reporting no DVA experience, after adjustment for confounders. This supports evidence attesting to the adverse health impacts associated with caregiving [14, 15], and with experiencing DVA [2, 3], respectively. Our findings also support two recent investigations of carers from different conditions, which reported significantly poorer health outcomes (e.g., elevated levels of depression, anxiety, stress and burden), in carers who experienced violence, compared to carers reporting no violence [42, 43].

### Accounting for differences in morbidities between carers and Non-Carers

Whilst acknowledging that power for interaction tests is often limited in epidemiologic studies [51] there was no evidence to support a modifying effect of DVA in the positive association between caregiving and health morbidities. This is in keeping with literature evidencing that DVA experience detrimentally affects the mental and physical health of victims in carer [42, 43], and general population samples [3]. After controlling for confounders, DVA experience and other adverse life events, caregiving remained a significant and independent predictor of CMD and poorer physical health. Experience of DVA was also significantly, independently associated with all measured health outcomes, after adjustment for confounders and other adverse life events. Our findings therefore confirm the adverse health impacts associated with experiencing DVA, both for carers and members of the general population. However, given the demonstrated elevated levels of DVA in carers, our findings suggest that addressing carers’ poorer health outcomes will also require identifying and addressing their DVA experience.

### Limitations

Approximately 15% of the sample identified with an ethnic group other than White British. Although consistent with the combined prevalence of these groups in the adult population resident in England, the Mixed/Multiple/Other ethnic category combined several distinct ethnic groups. The ethnic minority group categories were small and heterogeneous, with limited power for detailed comparisons across ethnic groups [29]. Furthermore, interviews were conducted in English; individuals with limited English proficiency were excluded. This supports the need for a boost of ethnic minority survey respondents, to enable more detailed analyses of findings across and within ethnic groups in future research [24]. Although self-completion modules were employed in parts of the survey, underreporting of stigmatised experiences (e.g., DVA), are still likely to occur [45]. Future research may benefit from considering how to sensitively approach discussions regarding carers’ experiences of violence [11], to encourage openness and confidence in having these discussions. It was not possible to confirm whether carers’ experiences of DVA were perpetrated by the care recipient or another relative, which precludes conclusions being drawn on the exact health impacts associated with experiencing care recipient violence, specifically. Exploring the family contexts which lead to, and the impacts associated with, carers’ victimisation, are warranted. Key characteristics of the caregiving role (e.g., duration of caregiving, types of care provided, care recipient condition), were not recorded. These characteristics can impact carers’ health outcomes [52, 53], and some are also associated with carers’ experiences of care recipient violence (e.g., care recipient illness) [42]. Future research would benefit from accounting for these characteristics, to afford a more detailed understanding of the associations between caregiving, experiences of violence, and health morbidities.

Finally, while broadly consistent with response rates of UK household surveys [54, 55], just over 40% of eligible respondents did not respond. Our results, therefore, should be interpreted with caution. It is conceivable that the pattern of results largely reflects the experiences of those who were the most impacted by their experiences in caregiving. Conversely, it is also possible that the carers most affected by their experiences were less likely to have engaged in the survey and therefore, the current findings underrepresent the full scale of the problem and carer need. Further, given the sampling window participants were asked to comment on their caregiving experiences and health status, participants with episodic mental health challenges and/or difficulties from an earlier time point would have been missed.

Notwithstanding these limitations, our findings offer implications for carer support strategies. Carers were more likely to be victims of DVA in adulthood than non-carers and these carers demonstrated significantly greater mental and physical health morbidities, than carers reporting no experience of DVA. Current NICE guidelines continue to outline that carers should be offered training to enable them to provide care safely, including training/support around managing challenging care recipient behaviour where appropriate [23]. However, professionals frequently encounter challenges in identifying and responding to DVA [56] Facilitating routine enquiry about DVA experience in carers will be essential [47], particularly given that carers may not readily and independently disclose their experiences of violence to professionals [11, 57]. Carers often delay seeking support for their own needs [58]. As every carer is entitled to a needs assessment [59], efforts to incorporate the identification of DVA experience into a carers needs assessment, which unpaid carers are entitled to [59] may help to facilitate identification, and referral to appropriate support services. The findings, consistent with other literature, also speak to the importance of clinicians optimising efforts to identify those in caregiving roles and ask about their caregiving experiences including specific information, support and care needs. This is particularly where carers might be exposed to and/or dealing with highly sensitive and challenging material such as domestic abuse and might be less inclined to or feel less comfortable in sharing their needs without being asked.

## Conclusion

Caregiving relationships and responsibilities have been evidenced to be a salient feature of domestic homicide reviews [10]. Yet the risk of victimisation in carers remains absent from current DVA policy in the UK [5]. Carers’ DVA victimisation is a sensitive and hidden topic [11] and, to date, its limited exploration as part of large representative samples is likely to contribute to a broader neglect of advancing our understanding within the literature and across health and social care of the impacts and implications of DVA and caregiving. Our findings underscore the importance of focusing and prioritising research efforts on the needs of informal carers.

## Data Availability

No datasets were generated or analysed during the current study.
